# Possible failure of novel direct-acting oral anticoagulants in management of pulmonary embolism: a case report

**DOI:** 10.1186/s13256-016-1135-9

**Published:** 2016-12-03

**Authors:** James Rankin, Menachem Nagar, Jonathan Crosby, Nojan Toomari, Richard Pietras, Uri M. Ben-Zur

**Affiliations:** 1 Division of Cardiology, Providence-Tarzana Medical Center, Tarzana, CA 91356 USA; 2 Department of Medicine, UCLA David Geffen School of Medicine, Los Angeles, CA 90095 USA

**Keywords:** Anticoagulant therapy, Warfarin, Factor Xa inhibitor, Thrombosis, Embolism, Case report

## Abstract

**Background:**

The relative effectiveness of vitamin K antagonists compared with novel oral anticoagulants in treating pulmonary embolism remains unclear. Recent trials comparing the efficacy of vitamin K antagonists with factor Xa inhibitors for the treatment of pulmonary emboli have been non-inferiority studies based primarily on risk reduction (such as bleeding events), rather than resolution of specific diseases such as pulmonary embolism. Consequently, there is a lack of evidence indicating which of these agents are more effective. Here, we present a case where pulmonary emboli were treated with novel oral anticoagulants followed by warfarin to discuss the potential limitations in the use of novel oral anticoagulants as prevention or treatment of thromboembolism and the continued role for warfarin in this setting.

**Case presentation:**

A 34-year-old African American woman presented to our clinic with shortness of breath and pleuritic chest pain several months post-surgery. She was identified as having multiple bilateral pulmonary embolisms and was treated with several novel oral anticoagulants, which failed to resolve the clots. Complete resolution was achieved upon switching to warfarin.

**Conclusions:**

The patient described in this report failed to respond to novel oral anticoagulant therapy, but her emboli resolved when she was treated with warfarin. This study challenges the notion that factor Xa inhibitors are better alternatives to vitamin K anticoagulants in the treatment of pulmonary emboli based on their safety profile and ease of use alone. As a result, further post-marketing investigations into the efficacy of these agents in the management of pulmonary emboli may be warranted.

## Background

Venous thromboembolism (VTE) is a condition that comprises deep vein thrombosis (DVT) with or without the presence of systemically circulating emboli; it includes conditions such as pulmonary embolism (PE). Prevention and treatment of VTE are imperative due to the significant morbidity and risk of mortality involved. Currently, agents available to treat this condition are limited and, among these, the mainstay of both prevention and treatment for over 60 years has been warfarin (Coumadin®; also Jantoven® and Marevan®), which is an oral vitamin K antagonist (VKA). However, warfarin treatment is inconvenient due to its increased risk of adverse events (that is, bleeding), which makes it an undesirable option [1, 2].

In response, new agents were developed to improve patients’ quality of life and to decrease the risk of bleeding in those requiring long-term anticoagulation. One class of these newer agents, the novel oral anticoagulants (NOACs) including direct factor Xa inhibitors (FXaI) and direct thrombin inhibitors, is quickly becoming the standard of care as first-line agents due to clear advantages with regard to monitoring and risks of adverse events. However, at this point, there is limited long-term data to determine all factors for their use in special circumstances. Here we present a case of a medication-compliant patient who experienced recurrent pulmonary emboli, yet failed to resolve these emboli on initial treatment with multiple NOACs, reaching complete resolution of emboli only after being switched to warfarin treatment.

## Case presentation

**Fig. 1:**
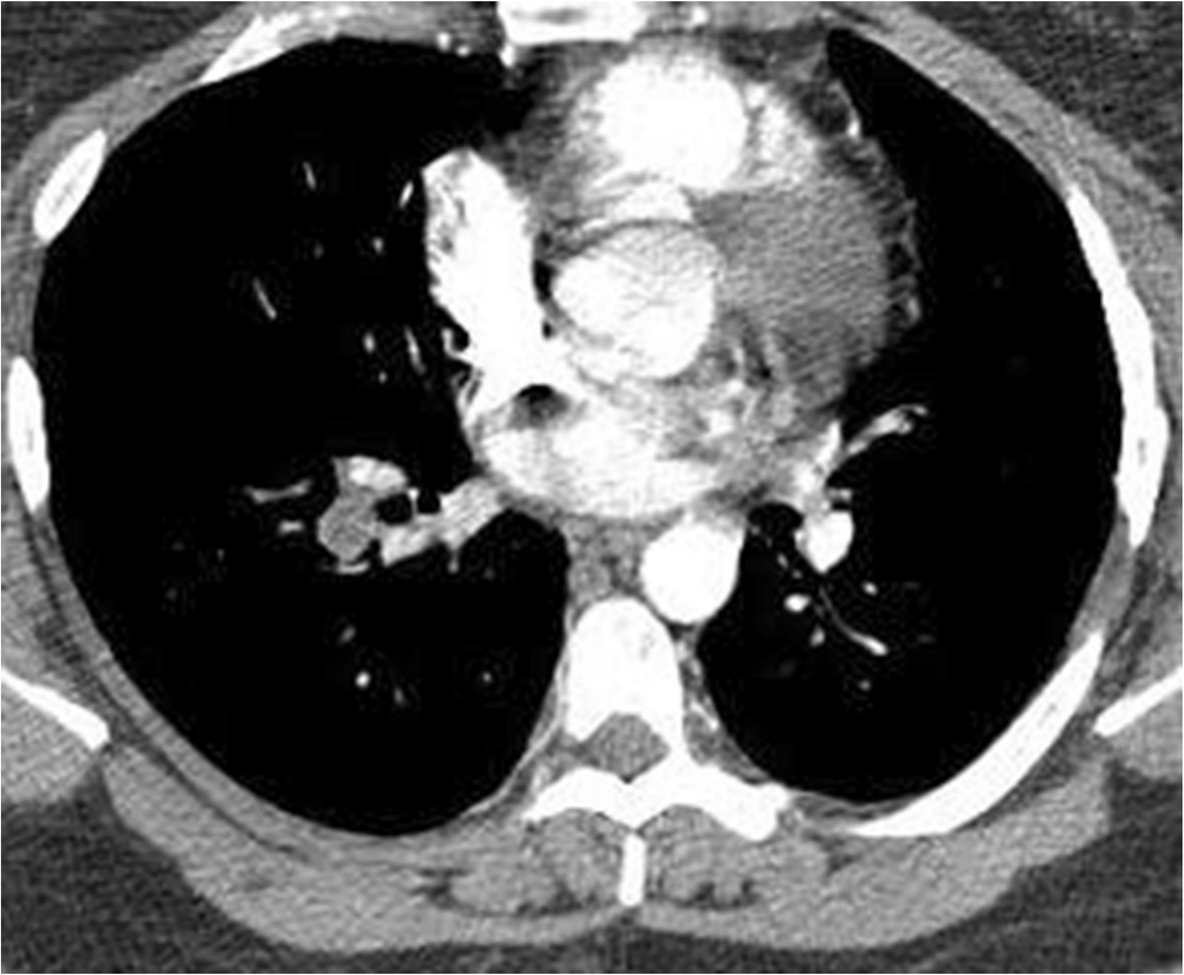
Computed tomography angiography of the chest with right-sided arterial emboli. Coronal section of this patient’s computed tomography angiography demonstrates a filling defect within the pulmonary vasculature, most likely representing emboli. At the time of this study, the patient was being treated with a novel oral anticoagulant (rivaroxaban)

Our patient is a 34-year-old obese (body mass index, BMI, 31.09 kg/m^2^) African American woman with a history of an undefined congenital septal defect, the status of which is post-surgical repair, and congenital clubfoot, who also underwent calcaneal tendon repair in May of 2015. Afterward, she remained sedentary for several weeks and then began to report progressively worsening non-exertional dyspnea that was associated with sharp intermittent chest pain. On 31 May 2015, she presented to her local emergency department for dyspnea on inspiration and “stabbing” pleuritic chest pain at bilateral lung bases. A computed tomography (CT) angiogram of her chest was performed, demonstrating multiple bilateral PEs (Fig. 1). She was then admitted to a hospital and started on rivaroxaban (Xarelto®, Janssen) dosed at 15 mg administered orally once a day. In September 2015, despite compliance with her rivaroxaban therapy for several months, a ventilation-perfusion (V/Q) scan demonstrated multiple bilateral pulmonary emboli. Rivaroxaban was discontinued and she was switched to dabigatran (Pradaxa®, Boehringer Ingelheim) 150 mg administered orally twice daily. Within a few days, she developed acute severe hip joint pain. She was then switched to apixaban (Eliquis®, Bristol-Myers Squibb) at 5 mg administered orally twice daily. On a follow-up V/Q scan in January 2016, she was found to have persistent PEs; she was then referred to our clinic.

**Fig. 2:**
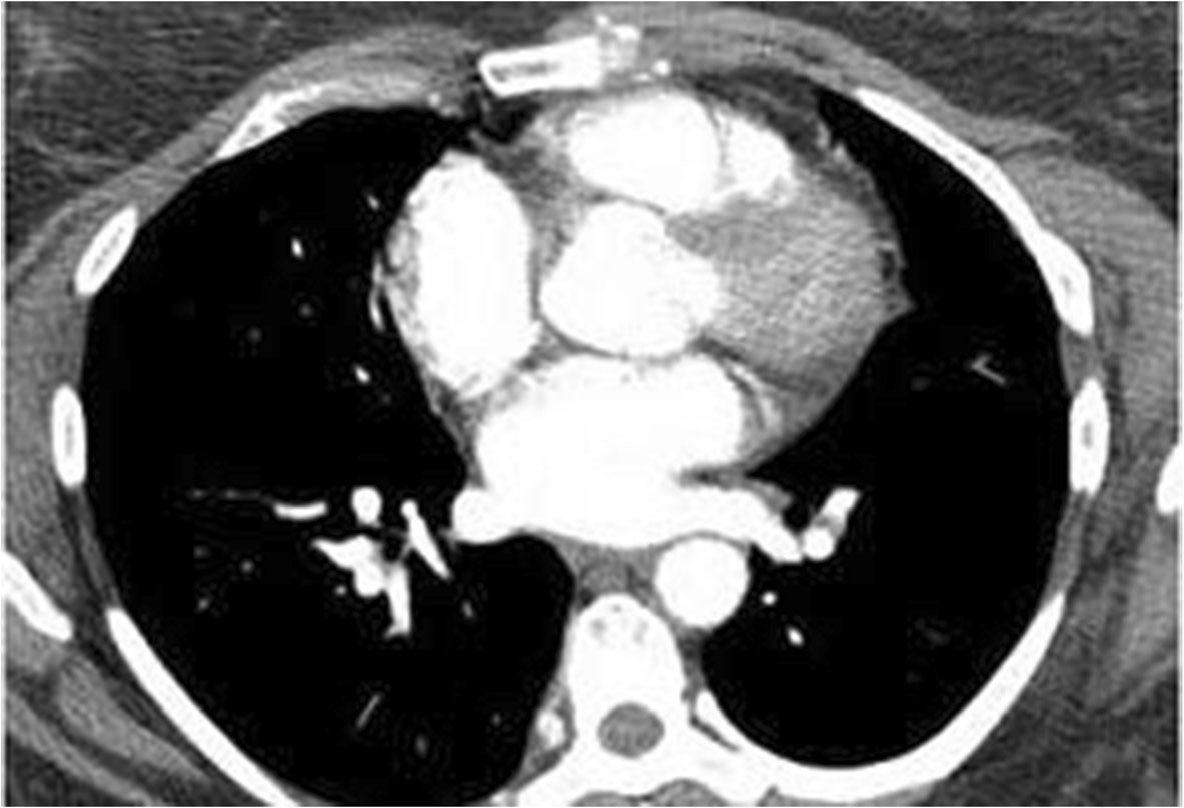
Computed tomography angiography of the chest without any evidence of emboli. A follow-up computed tomography after discontinuation of novel oral anticoagulant therapy and initiation of closely monitored conventional warfarin anticoagulation (international normalized ratio 2.0–3.0). A coronal section of this study at the same level as Fig. 1 reveals vessels filled with intravenous contrast indicating patency without the presence of emboli

Based on prior studies, apixaban was discontinued and warfarin therapy was initiated at 10 mg administered orally one a day with a goal of international normalized ratio (INR) 2.0–3.0, requiring weekly INR checks with appropriate dosing adjustments. She reported gradual progressive improvement and in March 2016 a CT angiography of her chest was performed with no evidence of pulmonary arterial thrombi or emboli (Fig. 2).

## Conclusions

For over 60 years, warfarin has been the standard out-patient management option for VTE. It has proven efficacy for this indication, significantly decreasing thromboembolic events while maintaining an acceptable level of adverse events, primarily bleeding episodes [1, 3]. This has been confirmed by numerous prospective studies and in post-marketing clinical use, including trials conducted to compare the efficacy of conventional doses of warfarin with low doses for providing anticoagulation. Conventional doses of warfarin (INR 2.0–3.0) were shown to provide superior treatment for VTE when compared with low-dose therapy (INR 1.5–1.9), notably with no significantly increased risk for "clinically important" bleeding [1]. However, warfarin use is not straightforward and requires: (a) bridging therapy (the use of parenteral anticoagulants until appropriate levels are attained), (b) frequent monitoring of individualized dosing to maintain INR levels within a narrow therapeutic window (in most instances, an INR goal between 2 and 3), and an (c) awareness of the significant risk of adverse bleeding events requiring potentially inconvenient lifestyle modifications [2]. Bleeding events associated with warfarin use range in severity from minor to fatal. One report indicated that warfarin elicited twice as many major bleeding episodes as placebo, while another found the incidence of major bleeding episodes in patients with a target INR greater than 3.0 to be twice as high as in those with a target between 2.0 and 3.0 [4, 5].

Alternatively, novel direct-acting oral anticoagulants aim to provide a safer, more tolerable, treatment option as compared to warfarin to prevent and treat DVT, PE, and atrial fibrillation (Afib) [3, 6]. Benefits of novel direct-acting oral anticoagulants include: (a) no need to monitor INR levels, and (b) potentially decreased drug interactions and adverse events (such as bleeding episodes). One family of novel direct-acting oral anticoagulants, the direct FXaIs, such as rivaroxaban (Xarelto®) and apixaban (Eliquis®), were only made available in the USA as recently as 2010 [7]. This family of drugs all share a similar mechanism of action, inhibiting the cleavage of prothrombin into thrombin during the final step of the common coagulation cascade. Several trials have compared FXaIs with warfarin in treating VTE. The EINSTEIN–PE trial showed rivaroxaban to be non-inferior to warfarin regarding PE treatment and demonstrated no difference in overall bleeding events [8]. Similarly, the AMPLIFY and Hokusai-VTE trials showed non-inferiority of apixaban and edoxaban, respectively, when compared with warfarin in the treatment of VTE, with a significant reduction in bleeding events [9, 10].

However, it is important to note that the trials reported to date have largely been non-inferiority studies, not equivalency or superiority studies. Data reported in the EINSTEIN–PE trial showed a downward trend in VTE episodes among those treated with warfarin versus rivaroxaban, but displayed a reduced number of bleeding episodes in the rivaroxaban group as compared with the warfarin group [8]. Most of these studies focused on treatment of VTE as a single category (DVT with or without non-fatal or fatal PEs), rather than reporting on the analysis of separate subsets (DVT, non-fatal PEs, fatal PEs, and so forth) contained within this larger group. Only one study, the EINSTEIN–PE trial, provided insight into the treatment of PE in isolation, revealing a trend toward superiority in warfarin-treated patients over FXaI in both non-fatal and fatal PE categories [8]. As a consequence of data available from these several recent trials, clinical guidelines, such as those of the “CHEST Guideline and Expert Panel Report”, now recommend use of newer agents, such as dabigatran, rivaroxaban, apixaban and edoxaban, rather than VKAs to manage patients with lower extremity VTE [11].

It is clear that non-inferiority studies are useful to determine the general safety profile of a novel therapeutic agent while retaining the efficacy of an accepted therapeutic standard. However, the lack of equivalency or superiority data and the absence of findings in established subsets of affected patients impede a clinician’s ability to critically evaluate what therapeutic agent may be best in treating specific conditions. Trials for new medications allow for the discovery and validation of agents, while decreasing potential risks, but these data should be regarded as tentative until more definitive studies have been performed in large populations, determining where the most benefit can be derived with the least possible risk.

It is accepted that common risk factors for clot formation include tobacco use, a history of coagulopathy, and/or the use of oral contraception. The patient in this report was counseled on these risk factors and denied either a history of tobacco use or clotting dysfunction; however, she was determined to be on levonorgestrel-releasing intrauterine system (Skyla®, Bayer). Although intrauterine devices are impregnated with hormonal contraceptive agents and carry a risk of thrombosis, Skyla was not reported to induce any cases during clinical trials, only as a part of post-marketing studies, and she developed clots after long-term use of this device [12].

Without significant retrospective analyses or randomized prospective trials with subgroup analyses, it is difficult to determine whether VKAs or FXaIs are truly more effective in the treatment of PE. This assessment is especially difficult when taking into account the diagnostic modalities and treatments provided for this patient prior to arrival in our clinic. Although she was initially followed with serial V/Q scans to determine the success of her treatments, the EINSTEIN–PE investigators and our clinic elected to use CT angiography in view of its substantially increased resolution [8]. In addition, prior to arrival in our clinic, she was treated with rivaroxaban at 15 mg administered orally once a day, as opposed to the generally recommended initial dose of 20 mg administered orally, followed by 15 mg administered orally twice a day [13]. In the case of rivaroxaban, in particular, these are important variables, because it is difficult to ascertain the effectiveness of a treatment if the standard of care is not being followed. However, the patient in this report was later managed with dabigatran and apixaban in accord with the recommended guidelines [14, 15].

We suggest that further investigations are needed to establish if novel direct-acting oral anticoagulants are equal to warfarin in the prevention and treatment of VTEs, including DVT and PE. Further, we suggest that practitioners carefully weigh the risks and benefits when selecting these medications. We note that Rudd *et al.* have reported independently on possible rivaroxaban failure in patients treated during the postpartum period, possibly due to pharmacokinetic alterations seen in the postpartum period that can contribute to decreased drug exposure and reduced anticoagulant efficacy [16]. In fact, rivaroxaban is well known to be metabolized by the cytochrome P450 isoenzyme CYP 3A4 and binds to P-glycoprotein; hence, leading to risks of pharmacokinetic interactions that may alter its anticoagulant properties [17]. In practice, it may be best at this time to choose between these several available anticoagulant drugs on a case-by-case basis, taking into account patient preferences, monitoring constraints, difficulty controlling the INR, the risk of bleeding and interactions, and the cost of treatment [18].

## Abbreviations

Afib: Atrial fibrillation
BMI: Body mass index
CT: Computed tomography
DVT: Deep vein thrombosis
FXaI: Factor Xa inhibitor
INR: International normalized ratio
NOAC: Novel oral anticoagulant
PE: Pulmonary embolism
VKA: Vitamin K antagonist
V/Q: Ventilation-perfusion
VTE: Venous thromboembolism
